# The potential of Virtual Reality as anxiety management tool: a randomized controlled study in a sample of patients affected by Generalized Anxiety Disorder

**DOI:** 10.1186/1745-6215-9-25

**Published:** 2008-05-05

**Authors:** Alessandra Gorini, Giuseppe Riva

**Affiliations:** 1 Applied Technology for Neuro-Psychology Lab, Istituto Auxologico Italiano, Milan, Italy; 2 Research Institute Brain and Behaviour, Maastricht University, The Netherlands; 3 Psychology Department, Catholic University of Milan, Italy

## Abstract

**Background:**

Generalized anxiety disorder (GAD) is a psychiatric disorder characterized by a constant and unspecific anxiety that interferes with daily-life activities. Its high prevalence in general population and the severe limitations it causes, point out the necessity to find new efficient strategies to treat it. Together with the cognitive-behavioural treatments, relaxation represents a useful approach for the treatment of GAD, but it has the limitation that it is hard to be learned. To overcome this limitation we propose the use of virtual reality (VR) to facilitate the relaxation process by visually presenting key relaxing images to the subjects. The visual presentation of a virtual calm scenario can facilitate patients' practice and mastery of relaxation, making the experience more vivid and real than the one that most subjects can create using their own imagination and memory, and triggering a broad empowerment process within the experience induced by a high sense of presence.

According to these premises, the aim of the present study is to investigate the advantages of using a VR-based relaxation protocol in reducing anxiety in patients affected by GAD.

**Methods/Design:**

The trial is based on a randomized controlled study, including three groups of 25 patients each (for a total of 75 patients): (1) the VR group, (2) the non-VR group and (3) the waiting list (WL) group. Patients in the VR group will be taught to relax using a VR relaxing environment and audio-visual mobile narratives; patients in the non-VR group will be taught to relax using the same relaxing narratives proposed to the VR group, but without the VR support, and patients in the WL group will not receive any kind of relaxation training. Psychometric and psychophysiological outcomes will serve as quantitative dependent variables, while subjective reports of participants will be used as qualitative dependent variables.

**Conclusion:**

We argue that the use of VR for relaxation represents a promising approach in the treatment of GAD since it enhances the quality of the relaxing experience through the elicitation of the sense of presence. This controlled trial will be able to evaluate the effects of the use of VR in relaxation while preserving the benefits of randomization to reduce bias.

**Trial Registration:**

NCT00602212 (ClinicalTrials.gov)

## Background

According to the DSM-IV-TR [1] the essential features of generalized anxiety disorder (GAD) are an excessive anxiety and an uncontrollable worry (apprehensive expectation), occurring more days than not for a period of at least 6 months, about a number of events or activities. Anxiety and worry are often accompanied by additional symptoms like restlessness, being easily fatigued, difficulty concentrating, irritability, muscle tension, and disturbed sleep. Although individuals with GAD may not always identify their worries as "excessive," they report subjective distress due to constant worry, have difficulty in controlling it, or experience related impairment in social, occupational, or other important areas of functioning. During the course of the disorder, the focus of worry may shift from one concern to another. Intensity, duration, or frequency of anxiety and worry are far out of proportion to the actual likelihood or impact of the feared event. Differently from other anxiety disorders usually associated to specific stimuli or situations, GAD is characterized by a constant and unspecific anxiety, involving a process of interacting systems (attentional, conceptual, imaginal, physiological, affective, and behavioural) that unfold over time in continual response to a constantly changing environment.

A quite recent study affirms that the prevalence of GAD in the general population might actually be as high as 5 to 8% [2], while among individuals seeing their physicians for psychological problems the 25% of them have a diagnosis of pure GAD. Indeed, in primary care facilities, GAD is the most frequent anxiety disorder and the second most frequent of all mental disorders [3,4]. GAD is more prevalent in women and much more prevalent in youths, where 12% to 20% are affected [5-7]. The age of onset is between the late teens and early twenties and the average duration is about 20 years, suggesting a chronic and fluctuating course that often worsens during time of stress. People with general nervousness, depression, inability to tolerate frustration, and feelings of being inhibited are more likely to be shown in GAD patients. People with GAD tend to have more conflicts with others, are very hard on themselves, and tend to avoid common situations for fear of worry and anxiety. In youths, GAD often leads to lower levels of social supports, academic underachievement, underemployment, substance use and high probability of developing other psychiatric disorders. In epidemiological studies of GAD a consistent finding has been that over 90% of individuals who meet criteria for GAD in a given year will also have at least one other DSM-IV diagnosis [8].

The high prevalence of GAD in the general population and the severe limitation it causes, point out the necessity to find new strategies to treat it in a more efficient way. GAD is usually treated with medications and/or psychotherapy. In particular, the two most promising treatments seem to be cognitive therapy and applied relaxation. As shown by numerous studies, both of these treatments are equally effective [9,10] immediately and over long-term periods. Cognitive treatment helps patients to recognize and alter patterns of distorted thinking and dysfunctional behaviour, while relaxation serves to reduce the increased physical arousal often strictly associated to this disorder. In fact, physical arousal can be voluntarily altered with training in relaxation skills that enables patients to shift physical functions voluntarily toward those that naturally occur in a relaxed state [11]. Progressive muscle relaxation and more general imagery techniques can be used as therapy progresses since the ability to relax, and to do it in any place or situation, is vital to reduce the anxiety level.

Even if relaxation represents a useful approach for the treatment of GAD, it presents an important limitation: it is difficult to be learned. Traditionally, relaxation techniques are verbally taught by a therapist or recorded on an audiotape, while recently a series of CDs of calming music have been used to help individuals to relax themselves, showing positive effects on anxiety reduction by achieving psychological benefits including distraction and sense of control over symptoms. Music interventions also have reported good results to reduce state and trait anxiety, to ease stress, and to increase relaxation [12-14]. These CDs strengthened the positive effect of calm and sedative music with relaxation techniques to achieve enhanced effects. To increase effectiveness, commercial relaxation DVDs have also integrated visual stimuli. In such a delivery, the visual representation of the scenario supports the process of relaxation creating an isolated context in which the subject can feel to stay.

Virtual reality (VR) can also be used to facilitate relaxation processes in stressed or anxious subjects [15] by visually presenting key relaxing images [16]. The advantage of VR compared to CDs or DVDs is its ability to induce a sense of presence in the users, that is defined as the "feeling of being in a world that exists outside of the self " [17]. The visual presentation of a virtual calm scenario can facilitate patients' practice and mastery of relaxation, making the experience more vivid and real than the one that most subjects can create using their own imagination and memory, and triggering a broad empowerment process within the experience induced by a high sense of presence. VR can be provided using desktops or laptops connected to a variety of peripheral devices, such as head-mounted displays and joysticks and even trough different kinds of mobile devices, such as the new generation of portable audio-visual devices, cellular phones and hand-held personal digital assistants (PDAs) equipped with enough raw horse power to deliver a good 3D experience.

### Aim of the study

Knowing that relaxation techniques are as effective as cognitive and behavioural methods in the treatment of GAD and considering the critical point regarding the difficult in learning them, the aim of the present study is to investigate the advantages of using a VR-based relaxation protocol in reducing anxiety in patients affected by GAD. The following hypothesis will be tested:

I) GAD patients exposed to a VR relaxing environment learn how to relax themselves easier and more efficiently than patients exposed to a traditional relaxation protocol;

II) There is a positive correlation between the treatment efficacy and the sense of presence experienced by the patients during the treatment sessions.

3-D audio-visual experiences, whose critical aspect is to induce a feeling of presence and engagement, will be presented to the subjects in order to help them to become relaxed. Through the link between the feeling of presence and the emotional state, VR relaxing environments will be used to reduce the anxiety levels in their users [15].

## Methods

The present trial is based on a randomized controlled study, including three groups of patients: (1) the VR group, (2) the non-VR group and (3) the waiting list (WL) group. Patients diagnosed with GAD according to the DSM-IV criteria, will be randomly assigned to one of these three groups.

### Subjects

Patients will be recruited in a public health-care structure in Milan, Italy, according to the diagnosis received from their treating responsible psychiatrist or psychologist. In order to be included in the study, patients have to meet the following criteria: (1) primary diagnosis of GAD; (2) requesting treatment for their GAD; (3) older than 17 and younger than 70; (4) no depressive disorder preceding the current episode of GAD or requiring immediate treatment; (5) no behaviour therapy received for their GAD; (6) no evidence of organic mental disorders accounting for the complaints, mental retardation, psychotic disorders, alcohol or drug dependence; (7) no migraine, headache, seizure disorder, and vestibular abnormalities since they represent significant contraindications for the use of VR.

A power analysis was conducted in order to evaluate the number of patients needed to detect a significant difference (Effect size f ≥ 0,40) between the three groups (VR, non VR and WL) with a power of 0,90. The analysis showed a necessary total sample size of 75, 25 in each group, with α = 0,5, two-tailed. Both males and females will be included. All participants have to be clinically stable, meaning no change in dose of psychotropic medications 1 month before inclusion. Since over 90% of individuals who meet criteria for GAD in a given year will also have at least one other DSM-IV diagnosis [8], patients will be included in the study even if they present at least one more psychiatric diagnosis.

Before participating in the study, each patient will be provided with written information about the study and invited to give written consent for inclusion. The present research is in compliance with the Helsinki Declaration.

### Withdrawal from the study

A patient must be withdrawn form the study when judged necessary by the responsible of the protocol or when the patient withdraws his/her informed consent.

### Clinical assessment

Subjects will be assessed by independent clinicians who will not be involved in the direct clinical care of any of them. They will be psychiatrists, MA-level chartered psychologists or PhD-level chartered psychotherapists. A semistructured interview will be used with the aim of identifying relevant DSM-IV diagnostic criteria for GAD in the subjects.

### Psychometric assessment

The following psychometric questionnaires will be administered to each patient at pre-treatment, upon completion of the clinical trial, and after 6-month and 12-month follow-up periods:

- PSWQ – Penn State Worry Questionnaire – [18]. The PSWQ is the only existing questionnaire specific for GAD with an extensive validation on Italian population. It analyzes symptoms of pathological worries that characterize this disorder.

- BAI – Beck Anxiety Inventory – [19]. The BAI is a standardised and well-used assessment measure with good psychometric properties used to assess anxiety. It is a 21-item scale which covers cognitive, behavioural and physiological symptoms of anxiety.

- Anti – Anxious Thoughts Inventory – [20]. The AnTI included 22 items that investigate 3 different categories of worries: social worries, worries linked to health and the metacognitions about worry (fear of fear). The available data show that the AnTI questionnaire is sensitive to treatment effects.

- CID – Anxiety and Depression Scales of the Clinical Interview for Depression – [21]. The CID anxiety scale consists of 4 items rated on a 7-point scale. The CID depression scale includes 10 items.

- STAI – State-Trait Anxiety Inventory – [22]. The STAI measures a person's situational (or state) anxiety, as well as the amount of anxiety a person generally feels most of the time (trait). The two scales contain 20 items each, which may be scored 1 (not at all) to 4 (very much so).

Immediately before and after each treatment session subjects will be also asked to rate their levels of subjective anxiety on the Visual Anxiety Scale (VAS-A). In order to verify if there is a positive correlation between the treatment efficacy and the sense of presence experienced by the patients during the treatment sessions, the Itc-Sense of Presence Inventory (ITC-SOPI) [23] will be administered to each patient exposed to the VR environment after each session. The ITC-SOPI is a questionnaire composed by 44 items, that investigates the sense of presence experienced during the VR exposure.

Moreover, as homework, patients will be asked to fill some forms regarding their relaxing experiences.

### Psychophysiological assessment

Immediately before and after each treatment session, psychophysiological parameters (skin conductance response (SCR), muscle tension, heart and respiratory rates) will be recorded in order to obtain objective measures of the emotional state of patients. Data will be recorded using the Procomp Infiniti Biofeedback system. The Procomp Infiniti System is an 8 channel, multi-modality encoder used to record different physiological parameters. Connected via USB to the computer, it records 8 parameters in real time and send them to the PC through fiber optic cables. In particular, in this study we are interested in recording 4 parameters: muscle tension, heart rate, respiration and skin conductance, using the following sensors:

#### • Electromyography sensor – EMG

The electromyography sensor, placed on the surface of the skin, directly above the forearm's muscles, detects small electrical current or signals that comes from active muscles registering the muscle tension.

#### • Blood Volume Pulse sensor – BVP

The BVP is a pulse detection sensor housed in a small finger worn package, to measure pulse rate of subject. The BVP sensor uses photoplethysmography to detect the blood pressure in the extremities. Photoplethysmography is a process of applying a light source and measuring the light reflected by the skin. At each contraction of the heart, blood is forced through the peripheral vessels, producing engorgement of the vessels under the light source, thereby modifying the amount of light to the photosensor. The resulting pressure waveform is recorded.

#### • Respiration sensor

The Respiration sensor is an easy fitting high durability latex rubber band fixed with velcro belt for monitoring respiration rate from a subject. The sensor is strapped to the torso to measure the relative amount of expansion that occurs during respiration (breathing). As breathing in takes place the rib cage expands which stretches the device. When exhaling, the stretch relaxes and the sensor returns to its neutral position. The resulting waveform is displayed on the screen.

#### • Skin Conductance Response sensors – SCR

**Figure 1:**
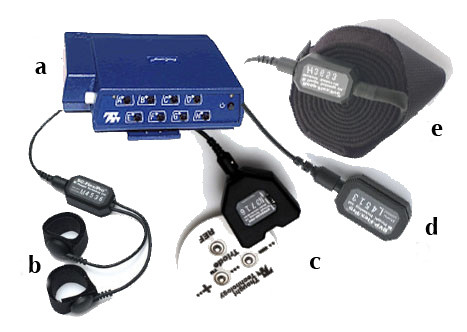
**The Procomp Infiniti System**. a) the Procomp Infiniti control box; b) the Skin Conductance Response sensors; c) the Electromyography sensor; d) the Blood Volume Pulse sensor; e) the Respiration sensor.

The Skin Conductance Response is a measure of the skin's conductance between two electrodes. Electrodes are small metal plates that apply a safe, imperceptibly tiny voltage across the skin. They are typically attached to the subject's fingers or toes using electrode cuffs or to any part of the body using a silver-Chloride electrode patch. To measure the resistance, a small voltage is applied to the skin and the skin's current conduction is measured. Skin conductance is considered to be a function of the sweat gland activity and the skin's pore size. An individual's baseline skin conductance varies for many reasons, including gender, diet, skin type and situation. Sweat gland activity is controlled in part by the sympathetic nervous system. When a subject is startled or experiences anxiety, there will be a fast increase in the skin's conductance (a period of seconds) due to increased activity in the sweat glands (unless the glands are saturated with sweat). After a startle, the skin's conductance will decrease naturally due to reabsorption. There is a saturation to the effect: when the duct of the sweat gland fills there is no longer a possibility of further increasing skin conductance. Excess sweat pours out of the duct. Sweat gland activity increases the skin's capacity to conduct the current passing through it and changes in the skin conductance reflect changes in the level of arousal in the sympathetic nervous system (see figure 1).

### Data collection

Epidemiological, clinical and all outcome data from each patient will be recorded by the clinician responsible of the study in an electronic database. Psychophysiological data from the Procomp Infinity System will be transformed and recorded in Excel files.

At the end of the treatment, clinicians will arrange a 6 and 12-month review appointment with the patients in the intervention groups and a 12-month review for those in the WL group. At those points clinical follow up data will be collected. A reminder of forthcoming appointments will be distributed to each participant practice 2 weeks before the appointment is due.

### Experimental design

Patients included in the study will be randomly assigned to one of the following three conditions:

(I) VR: subjects will be taught to relax using VR relaxing environments and audio-visual mobile narratives;

(II) non-VR: patients will be taught to relax using the same relaxing narratives proposed to the VR group, but without the VR support;

(III) WL: patients included in the waiting list group will not receive any kind of relaxation training.

Psychometric and psychophysiological outcomes will serve as quantitative dependent variables, while subjective reports of participants will be used as qualitative dependent variables.

### Randomization

The present trial is a randomized controlled trial. Patients will be randomly assigned to one of the three groups of treatment using numbers generated at random [24].

### Hardware

**Figure 2:**
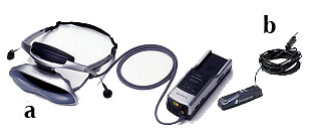
**The virtual reality equipment**. a) the Head Mounted Display, Sony Glasstron PLM S-700; b) the position tracker, Intersense Intertrax2 256 Hz.

The VR system is composed by (see figure 2):

- a laptop (Asus G2S; Intel^® ^Core™2 Extreme Processor X7800);

- an Head Mounted Display (HMD), Sony Glasstron PLM S-700, equipped with a visual device for a 3D view of the virtual environment and an audio device (earphones) to listen to the narratives;

- a position tracker, Intersense Intertrax2 256 Hz, that allows the user to modify his/her point of view in the virtual environment according to his/her movements in the real world;

- a joystick.

The iRiver Clix, a portable audio-visual device, will be assigned to each patient included in the two experimental groups, in order to help him/her to practice the relaxation techniques out of the therapist's office. Patients in the VR group will use it to play video and sound, while the others will use it just to listen to the narratives.

### The virtual environment software

In the present protocol we will use two different virtual environments included in the open-source software NeuroVR (version 1.5) [25].

**Figure 3:**
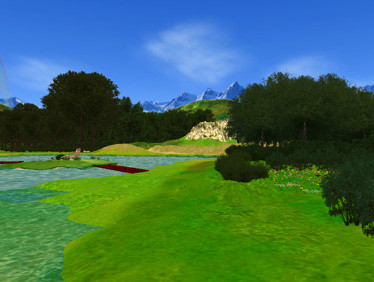
**A screenshot from NeuroVR (V. 1.5)**. The Green Valley is the virtual environment used to facilitate relaxation in the VR group.

For the relaxation sessions, the Green Valley, a very relaxing environment showing a mountain landscape around a calm lake is presented together with the relaxing narrative and soft sounds (birds' songs, water flowing, etc) (see figure 3). Patients are asked to walk around the lake, to observe the nature and, after few minutes, to virtually seat on a comfortable deck chair, in order to become easily relaxed.

**Figure 4:**
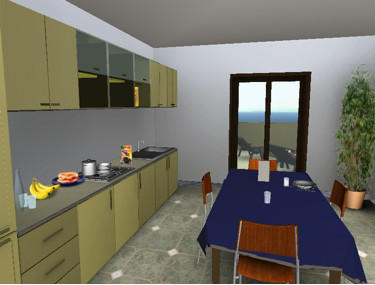
**A screenshot from NeuroVR (V. 1.5)**. The figure illustrates a virtual kitchen, one of the stressful environments showed to the patients during the last week of treatment.

For the second part of the protocol, patients will be presented with specific stressful virtual environments that simulate real-life situations: a crowded place, a classroom, an apartment, and so on (see figure 4). Each of these environments can be modified by the therapist with objects, persons and multimedial components depending on the patients' needs.

### Clinical protocol

The clinical protocol will be based on 7 weeks of Applied Relaxation (AR) training (2 sessions per week). Patients will be taught coping skills which enable them to relax rapidly, in order to counteract and possibly eliminate the anxiety reactions.

We will now describe the protocol in details, following the one developed by Ost in 1987 [26]. The sessions will be the same for both groups (VR and non-VR), with the only difference that the VR group is presented with audio narratives accompanied by virtual relaxing environments, while the non-VR group only hear the narratives.

The protocol will follow the following schema:

- Session one: the therapist clearly explains the patient the role of bodily arousal in the maintenance of anxiety and underlines the fact that relaxation procedures can be useful to reduce the autonomic arousal. A general description of the method used in the following sessions will be given to the patient. After an oral presentation, patients will receive a short written description of the rationale. In this way, they will have the possibility to read the text as many times as they want, to ask questions about unclear points and to study it at home.

The following is an example of the text given to the patients (adapted from Ost, 1987):

"When a person suffers from an excessive anxiety usually manifests three different components in his/her reaction: physiological (increased heart rate, blood pressure, sweating etc.), behavioral (trying to escape from the anxious situation, trembling etc.), and subjective (negative thoughts like 'I am going to faint or lose control' etc.). The strength of these components varies between patients, but previous research has found that most people experience some physiological changes, followed by a negative thought, which increases the physiological reaction, generating a vicious circle.

One good way of breaking this development is to focus on the physiological reactions and learn not to react so strongly. The method we are going to use to achieve this is called applied relaxation. The aim of this technique is to learn a skill of relaxation, which can be applied very rapidly and in any real-life situation. This skill can be compared to any other skill, e.g. learning to swim, ride a bike, or drive a car, in that it takes time and practice to be learned, but once you have mastered it, you can use it anywhere. You are not restricted to the calm and non-stressful situation in the therapist office or your own home.

The goal of the treatment is to be able to relax in few sessions and to use this skill to counteract, and eventually get rid of, the physiological reactions you usually experience in anxious situations. To achieve this we are going through a gradual process starting with tensing and relaxing different muscle groups.

The next step teaches you to connect the self-instruction 'Relax' to the bodily state of relaxation. After that it is time for the rapid relaxation, which you practice many times a day in non-stressful situations. Finally, you reach the stage of applying the skill in anxious situations, and I will take you to different anxiety-arousing situations coaching you how to apply the relaxation at the first signs of anxiety in these situations. Applied relaxation is thus a skill that most people can acquire with the right instructions and practice. It is a 'portable' skill that can be used in almost any situation, e.g. when having problems in falling asleep.

During the entire protocol you'll be guided by relaxing narratives (and images) that suggest you how to reach a relaxed state".

The second part of the session one will be dedicated to fill the following questionnaires: PSWQ, BAI, AnTi, CID and STAI.

The following sessions will be dedicated to teach patients to recognize early signals of nervousness and to cope with anxiety. Each session lasts about one hour and is divided in four parts: homework checking, relaxation, comments about the experience (debriefing) and new homework assignments. Immediately before and after the relaxation phase physiological and psychometric measures will be taken in order to measure the objective and subjective effects of relaxation on the patient. The relaxation setting consists in a very quite room scarcely enlighten in which the patient sits in a comfortable armchair.

Sessions 2–5: during these sessions different narratives [27,28] guide patients in muscle relaxation. Using the "Emotioneering" approach [29], in the VR group the relaxing narrative will be linked to the presentation of the Green Valley.

Sessions 6–9: in these sessions the focus is on the breathing other than on the muscle relaxation. Again, in the VR group, the relaxing narrative will be linked to the presentation of the Green Valley.

Sessions 10–13: after nine sessions and weeks of homework practice, patients are ready to start applying the relaxation skill in stressful situations. During these last sessions a gradual exposure to feared situations is introduced so that patients can practice their applied relaxation skills. The exposure will be different for the two experimental groups. The VR group will be presented a virtual environment representing one of the situation that most frequently elicits anxiety in the patient, while patients in the non-VR group will use imaginal exposure to imagine real-life situations that usually provoke high level of anxiety.

Session 14: this session is devoted to the final debriefing. The patient and the therapist discuss about the results obtained with relaxation. Patients are encouraged to develop the habit of practice relaxation at least once a day. They are also encouraged to record their continued practice on a specific form during the first 6 months after the end of the treatment and to mail it regularly to the therapist.

**Figure 5:**
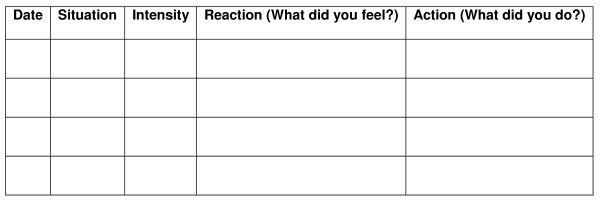
Form of self-observation of early anxiety signals (adapted from Ost, 1987).

**Figure 6:**
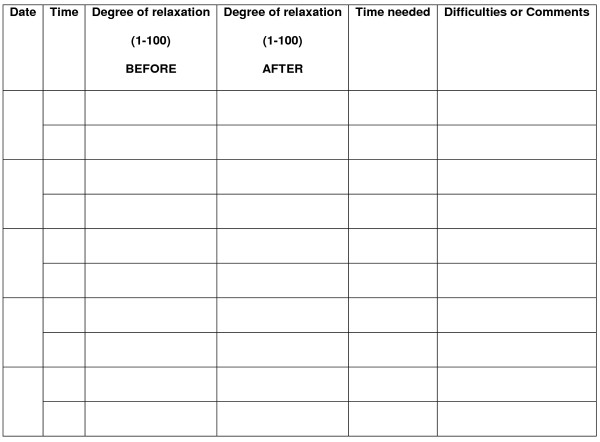
Form for registration of homework relaxation training (adapted from Ost, 1987).

During the entire treatment, patients are given different kind of homework assignments useful to increase their awareness during the every-day life and not only in the therapist's office. The first assignment consists in self-observation: patients are asked to record their anxiety reactions and to recognize and describe each situation that provokes them. In particular, between the sessions 2 and 5 patients are asked to fill a form including only Date, Situation and Intensity of the anxiety reaction (see figure 5). Between the sessions 6 and 9 they also have to fill a column called "Reaction" (that means: What did you fell?) and between the sessions 10 and 13 another column called "Action" (What did you do?) will be also included. The second assignment consists in practicing relaxation at least once a day using the portable audio-visual device they have received from the therapist. Every time they practice relaxation, they are asked to fill another form registering at what time they have practiced, how relaxed they were before and after the practice, how long it took them and eventually which difficulties they have experienced (see figure 6).

### Follow up assessment

Six and twelve-month follow-up are planned in order to verify the efficacy of the treatment over a long term period.

### Potential benefit of VR

The use of VR will be introduced with a goal of increasing the subjects' ability to relax themselves easier than they do using the traditional relaxing methods. If analyses find that the use of VR improves the relaxation processes and the reduction of anxiety, then a range of benefits are possible. For example, satisfactory ability to manage the patient's anxiety could be increased and length of treatment could be reduced.

### Potential harms of VR

Potential for harms is minimal. The only risk really associated with the use of VR is the so-called cybersickness, that is characterized by symptoms that parallel those of classical motion sickness both during and after the virtual experience. Cybersickness is rare and sometimes disappears after few minutes of rest.

### Trial analysis

Data will be entered in SPSS. Descriptive methods will be used to demonstrate the consistency of the three groups, describe participant characteristics and report levels of participation and drop out. Analysis of variance will be used to evaluate baseline characteristics of the three groups involved in the study, overall significance of improvement across outcome measures, and drop out versus maintainers. For each patient, change in psychometric and physiological measures will be calculated and analysed using t-test for matched groups for both intervention and control groups. The magnitude of change will be estimated and the 95% confidence intervals given.

## Discussion

Despite a large number of studies showing the advantages of using VR for the treatment of anxiety disorders (for a recent review see [30]), up to date there is only one study about its potential in stress management and relaxation processes in a sample of non-clinical stressed subjects [15]. What we would like to show with the present study is that VR can facilitate the reduction of anxiety even in a sample of GAD patients. We believe that the use of immersive VR for relaxation represents a promising approach since it enhances the quality of the relaxing experience through the elicitation of the sense of presence. In fact, as reported by different studies [31-34], individuals who interact in environments enriched with a variety of positive visual and auditory stimulations, report greater improvement in self-efficacy and mood. Being able to become relaxed in a certain situation promotes the sense of self-efficacy and prepares participants to cope with real anxiety situations.

Our outcome measures are well-suited to measuring both the subjective and objective impact related to the intervention and the presence of a non-VR group and of a WL list group will guarantee that the results eventually found are really attributable to the intervention. This controlled trial will be able to evaluate the effects of the use of VR in relaxation while preserving the benefits of randomization to reduce bias. Its design takes into account the need for internal and external validity and that the results are attributable to the intervention. Since the international scientific community is now starting to widely recognize the potential of VR as clinical tool [35] we look forward to adding the results of this trial to the growing body of literature in this area.

## Abbreviations

GAD – Generalized Anxiety Disorder; VR – Virtual Reality; WL – Waiting List group; PSWQ – Penn State Worry Questionnaire; BAI – Beck Anxiety Inventory; Anti – Anxious Thoughts Inventory; CID – Anxiety and Depression Scales of the Clinical Interview for Depression; STAI – State-Trait Anxiety Inventory; VAS-A – Visual Anxiety Scale; EMG – Electromyography; BVP – Blood Volume Pulse; SCR – Skin Conductance Response

## Competing interests

The authors declare that they have no competing interests.

## Authors' contributions

Both authors participated in the conception and design of the study. AG was involved in drafting the manuscript, while GR made a critical revision of it. Both of them read and approved the final version of the manuscript.
